# Estimating a Logistic Discrimination Functions When One of the Training Samples Is Subject to Misclassification: A Maximum Likelihood Approach

**DOI:** 10.1371/journal.pone.0140718

**Published:** 2015-10-16

**Authors:** Nico Nagelkerke, Vaclav Fidler

**Affiliations:** 1 Malawi-Liverpool-Wellcome Trust, Chichiri, Blantyre 3, Malawi; 2 Haren, Groningen, The Netherlands; University College London, UNITED KINGDOM

## Abstract

The problem of discrimination and classification is central to much of epidemiology. Here we consider the estimation of a logistic regression/discrimination function from training samples, when one of the training samples is subject to misclassification or mislabeling, *e*.*g*. diseased individuals are incorrectly classified/labeled as healthy controls. We show that this leads to zero-inflated binomial model with a defective logistic regression or discrimination function, whose parameters can be estimated using standard statistical methods such as maximum likelihood. These parameters can be used to estimate the probability of true group membership among those, possibly erroneously, classified as controls. Two examples are analyzed and discussed. A simulation study explores properties of the maximum likelihood parameter estimates and the estimates of the number of mislabeled observations.

## Introduction

The problem of discrimination and classification has generated an extensive literature both from the biostatistical/epidemiological and from the machine learning communities. In its simplest form two populations, identified by a binary (dependent) variable y (y = 0 for controls or non-cases and y = 1 for cases, *e*.*g*. individuals with a specific disease), have to be distinguished on the basis of a set of p (*e*.*g*. genetic or behavioral) traits or (independent) (co)variables **x** = (x_1_,..,x_p_)^T,^. In addition, the role of individual components of **x** is often also of interest as this may provide insight in the mechanisms that generate values of y; for example the role of a (mutant) gene in the etiology of a disease. A discrimination rule has to be estimated on the basis of a training sample of n = n_0_+n_1_ observations, representative of the two underlying populations; in machine learning terminology this is called supervised learning. Solutions range from classical Fisher Linear Discriminant Analysis and Logistic Regression to kernel based methods, Random Forests and Support Vector Machines. A nice overview is given by James *et al* [1].

A problem that, while surprisingly common in practice, has apparently attracted limited attention, is how to develop a discrimination rule when training samples have been “contaminated” by misclassification or mislabeling of the group membership. This type of misclassification is also known as “label error” or “label noise”. Mislabeling, that is misclassification of labels (*i*.*e*. the outcome or dependent y variable in the context of binary regression) may arise, for example, because the labeling involves some guesswork or subjective judgment as in medical diagnoses. The presence of misclassification/mislabeling in training samples is often ignored in practice, or the problem is redefined in terms that fit available solutions. When researchers are aware of the problem it is also sometimes dealt with by removing or relabeling observations, either in an *ad-hoc* heuristic manner, or using an algorithm such as “depuration” that essentially removes “outliers” [2].

Some authors who explored this problem more formally were Lugosi[3], who explored this problem in the context of non-parametric pattern recognition, and Manevitz and Yousef [4], who explored this problem when only observations from one (sometimes misclassified) population are available. Some authors treated the “true label” as a latent or missing variable and used the EM algorithm for parameter estimation [5–6]. Some other authors considered the related problem of developing a discrimination rule when observations with a set of labels are available, only one of which is correct [7]. This situation of course is less relevant to the common situation when only two labels are possible. In fact, in this situation, *i*.*e*. no information about the true label of any observation is available, the problem becomes a mixture analysis problem. Some authors considered the two possible labels mislabeling problem in the context of an underlying logistic regression function or discrimination rule. This is highly relevant for epidemiological case-control studies, where other types of discrimination rules cannot be estimated from this type of stratified samples. Nagelkerke *et al* considered this in the context of “mixture” situations where no true labels are available [8]. As early as 1966 Lachenbruch explored the effect of mislabeling on Fisher's discriminant functions (also logistic functions), and found that, to first order, the effect is to shrink all coefficient towards the null with the same coefficient K and concluded that if some members of the original samples are incorrectly classified the utility of the discriminant function may not be seriously affected [9]. This however, is not relevant if interest is in the values of the coefficients, or if the probability of the true label is important, as is the case in medical decision problems. For example, correctly estimating the probability of seminal vesicle involvement in prostate cancer is key in making optimal therapeutic decisions. More recently, Albert *et al* [10] considered this mislabeling problem in situations in which there is a subsample with known true case status. Bootkrajang and Karan thoroughly addressed the mislabeling problem in the context of logistic discriminant rules in its most general form and explored, as we shall do for the one-sided (misclassifications occur in one direction only) problem, likelihood estimators for the parameters of the underlying logistic function [11–13].

Here we want to discuss the situation when only one of the training samples, in particular the control sample (but this is arbitrary of course), contains a non-negligible proportion of the other group (here cases). We assume that the underlying true discrimination rule–in which we are interested–is a logistic one.

The motivating example comes from public health, genetics, and microbiology. Legionnaires disease is a serious bacterial (Legionella Pneumophilia) infectious disease, that results from inhaling contaminated aerosols, *e*.*g*. generated by an air conditioning installation or a Jacuzzi. The bacteria are almost ubiquitous but not all strains of the bacteria are pathogenic. To develop a test for the pathogenicity of a strain, on the basis of the genome of the bacteria, one should ideally compare pathogenic strains, isolated from patients, with non-pathogenic control strains. However, such control strains clearly do not exist, and instead environmental samples, comprising of a mixture of non-pathogenic and pathogenic strains, are used as controls.

This one-sided mislabeling problem however is very common. In data from health surveys, one can only compare people reporting hypertension, almost all of whom probably having this condition, to those who do not. A substantial proportion, however, of hypertensives may not be aware of their condition and are thus mislabeled as normotensives. In criminology, one can easily compare individuals convicted of a specific crime (say burglary) to those who have not, although many burglaries never result in convictions. Another common situation where this occurs is in the context of determining the sensitivity and specificity of a (new) test, when the putative gold standard itself has imperfect specificity or sensitivity. This is the case, for example, in tuberculosis, where culture is traditionally considered the gold standard, although there may be false-negative culture results [14]. Ignoring this problem may often be undesirable as in our legionella example where identifying strains as pathogenic may lead to costly public health interventions. It also has implications for the performance of classification methods. For example, boosting methods that iteratively increase the weight of misclassified observations may not work properly, and thus should either be avoided or applied asymmetrically, as argued by Long and Servedio [15].

## Methods

Consider a population of stratified training samples, z = 0 and z = 1, sampled in the proportions n_0_:n_1_ from the populations of possibly contaminated controls and cases. Denoting by y = 1 the true cases and by y = 0 the true controls, we have P(y = 1|z = 1) = 1 and P(y = 1|z = 0)≥0, that is all observations with z = 1 are actually y = 1, but a possibly positive fraction of those with z = 0 is actually y = 1. Consequently, there will also be a positive probability λ = P(z = 0|y = 1,**x**) (assumed to be independent of **x**). This probability depends both on P(y = 1|z = 0), which is assumed to be a fixed parameter, and the population mixing proportion n_0_:n_1_.

Here we consider this problem in the context of logistic regression. That is we assume that

$$P(y=1|x)=\exp(\beta_{0}+\beta^{T}x)/(1+\beta_{0}+\beta^{T}x)$$

where **β** = (β_1_,…,β_p_)^T^ and β_0_ obviously depends on the proportion of true cases (y = 1) in the total sample. For the probabilities of z conditional on **x** we have

P(z=1|x)=P(z=1|y=0,x)⋅P(y=0|x)+P(z=1|y=1,x)⋅P(y=1|x)

which in the case of an underlying logistic regression function becomes

$$\left(1-\lambda )\cdot \exp(\beta_{0}+\beta^{T}x)/(1+\beta_{0}+\beta^{T}x\right)$$

Similarly,

P(z=0|x)=P(z=0|y=0,x)⋅P(y=0|x)+P(z=0|y=1,x)⋅P(y=1|x)P

which again, by assuming a logistic regression function, becomes

$$\left[1+\lambda \cdot \exp(\beta_{0}+\beta^{T}x)\right]/(1+\beta_{0}+\beta^{T}x)$$

As P(z = 1|**x**) assumes values between 0 and 1-λ, it can be termed a *defective* logistic regression (DLR). This model is formally equivalent to the zero-inflated logistic regression model, see *e*.*g*. Hall [16]. Although (unlike zero-inflated count models) this model appears to be unavailable in major statistical packages, similar to standard logistic regression, parameters of this function can be estimated easily using maximum likelihood. In cases where, unlike in our examples, the number of covariables p is large compared to n_0_+n_1_ estimation (regularization) methods penalizing model complexity, *e*.*g*. the lasso which penalizes the absolute values of the β coefficients (the λ should perhaps not be penalized), should be used (we did not as we considered only few covariables).

As P(y = 1) = P(z = 1,y = 1)+P(z = 0,y = 1) = P(z = 1) + λ∙P(y = 1), we have P(y = 1) = P(z = 1)/(1-λ). Thus the expected number of cases in the total sample equals n_1_/(1-λ), and the expected number of controls who are actually cases is n_1_∙λ/(1-λ).

The parameters β_0_, **β** in the defective logistic regression model are identical to the ones in the underlying logistic model for y. Thus the latter model can be used for classification. (As β_0_ depends on the arbitrary mixing proportions of cases and controls these probabilities should be interpreted cautiously.). As our objective is the prediction (classification) of the y (instead of the observed, but possibly mislabeled, outcomes z) values of the observations, techniques such as cross-validation to explore the performance of our proposed method in given empirical contexts, are not meaningful. Cross-validation would require a subset of observations, not used in estimating the classification function, for which the true, or gold standard, outcomes are known. This is obviously not the case.

## Results

We apply our proposed method to two examples. In both examples we used the mle() function from the stats4 library of the statistical package R [17] for ML estimation of the DLR parameters. The loglikelihood, as usual, is the sum over all observations of {zlog(P(z = 1|**x**)) + (1-z)log(P(z = 0|**x**)}. Use of the maximum likelihood guarantees that–if the model is correctly specified–the parameter estimators have well established nice properties such as consistency and (asymptotic) efficiency [18]. Also, criteria such as AIC, BIC, significance testing, etc. for deciding which elements of **x** to include in the (final) model, can be used. However, we advise to base the decision to use defective logistic regression instead of standard logistic regression on prior, substantive, knowledge that non-negligible mislabeling occurs. This is, because standard asymptotic theory makes the assumption that the true parameter value λ lies away from the boundary λ = 0.

We used the solutions of the standard logistic regression, that is the maximum likelihood solution when fixing λ = 0, as starting values. These starting values will guarantee convergence to a (possibly local) maximum with a likelihood at least as large as that of the standard logistic model. It is also possible to plot the profile likelihood of λ (*i*.*e*. the maximum likelihood–over β-for given values of λ) to ensure that the likelihood is well-behaved in the relevant region of the parameter space. Where appropriate, as in example 2, we also bounded λ by 0 below and by n_0_/(n_0_+n_1_) above using the L-BFGS-B method.

**Fig 1:**
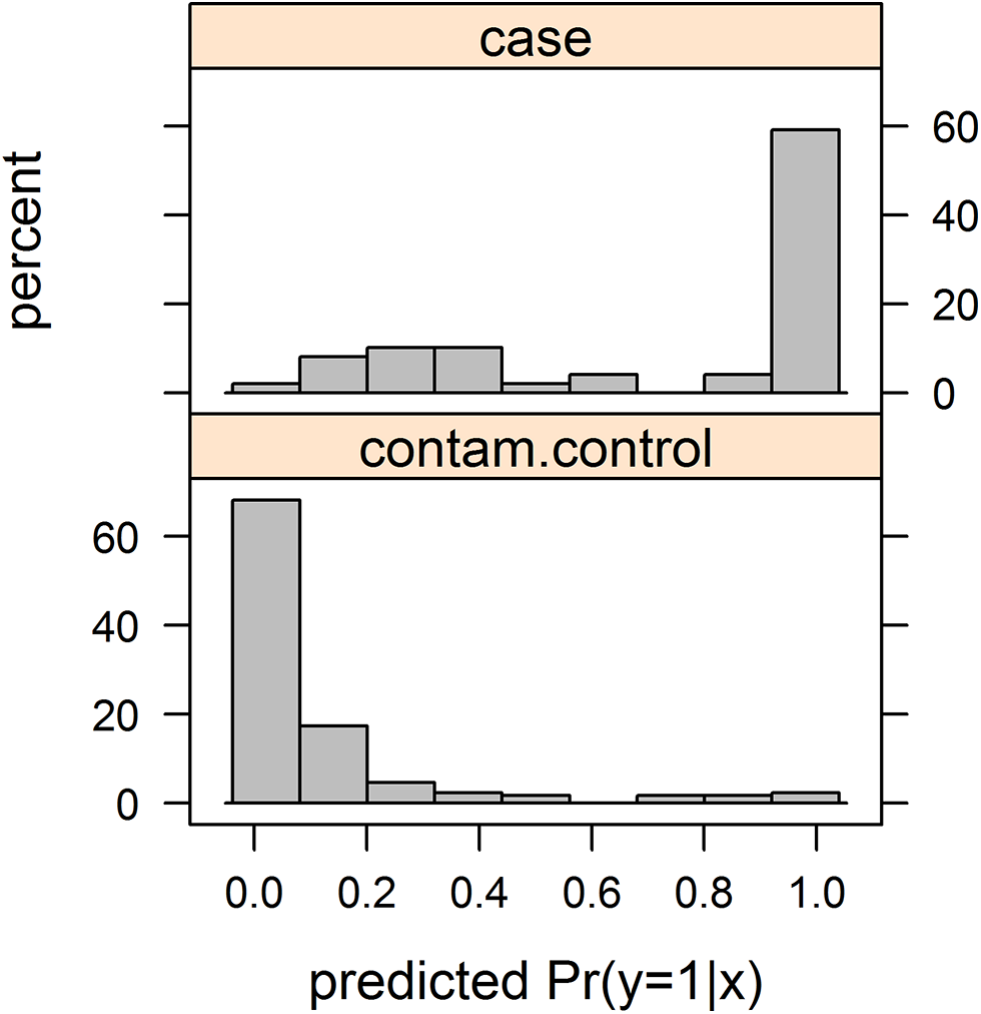
Frequency distribution of predicted probabilities of being a case in Example 1.

As our first example, we consider the above described problem of identifying pathogenic legionella strain when the environmental control sample potentially contains several pathogenic strains. In short 49 pathogenic strains are to be compared to 173 environmental ones. We restricted ourselves to the four genetic markers previously identified as important by Euser *et al* [19]. Table 1 summarizes the data, while Table 2 presents results of fitting the DLR and logistic regression (LR) model. We reparameterized λ to μ = λ/(1-λ) because it yielded greater symmetry of the profile likelihood. The ML estimate of the expected number of environmental strains which are actually pathogenic, n_1_ λ/(1-λ), is 9 (95% CI: 0 to 19). There seems to be a substantial difference for the third marker, justifying further exploration of its role in pathogenesis. The likelihood ratio test (the difference in twice the log-likelihood between the defective logistic regression and usual logistic regression models) yields P = 0.078, the Wald test statistic for testing the null hypothesis λ = 0 gives P = 0.047. Thus the DLR model fits slightly better than the LR model. Using the DLR estimates of β_0_ and β we calculated the predicted probabilities P(y = 1|**x**) of being a case, and also P(y = 1|x,z = 0). These probabilities can be used for classification choosing a suitable cut-off. (As β_0_ depends on the chosen proportions of pathogenic and environmental strains, these probabilities should be interpreted cautiously). As an example we chose a cut-off 0.285 for which 9 of 173 contaminated controls were classified as cases (9 is the estimate by the fitted DLR model), that is the DLR model estimated probability P(Y = 1|z = 0,x) exceeded 0.285. Then, also, the estimated P(y = 1|x) of 41 of 49 cases (*i*.*e*. z = 1) exceeded the cut-off. Histograms of estimated probabilities P(y = 1|x) are shown in Fig 1.

**Table 1:** Summary statistics of the 4 genetic traits in the 49 pathogenic and 173 environmental legionella strains.

	Environmental strains	Pathogenic strains
Trait	Mean (SD)	Mean (SD)
L07B8	.42 (2.11)	8.54 (8.24)
L15D6	.34 (.43)	.81 (.56)
L16E4	.99 (.34)	1.30 (.17)
L33F8	1.13 (.29)	.83 (.44)

**Table 2:** Parameter estimates and their standard errors of DLR and LR estimation.

	DLR		LR	
variable	estimate	SE	estimate	SE
μ = λ/(1-λ)	0.180	0.108		
b_0_	-5.255	2.240	-4.200	1.325
b_L07B8_	0.609	0.506	0.187	0.050
b_L15D6_	0.984	0.567	0.984	0.412
b_L16E4_	4.865	1.885	3.430	0.967
b_L33F8_	-2.566	1.087	-2.060	0.663
-2∙log(L)	122.57		125.67	

**Fig 2:**
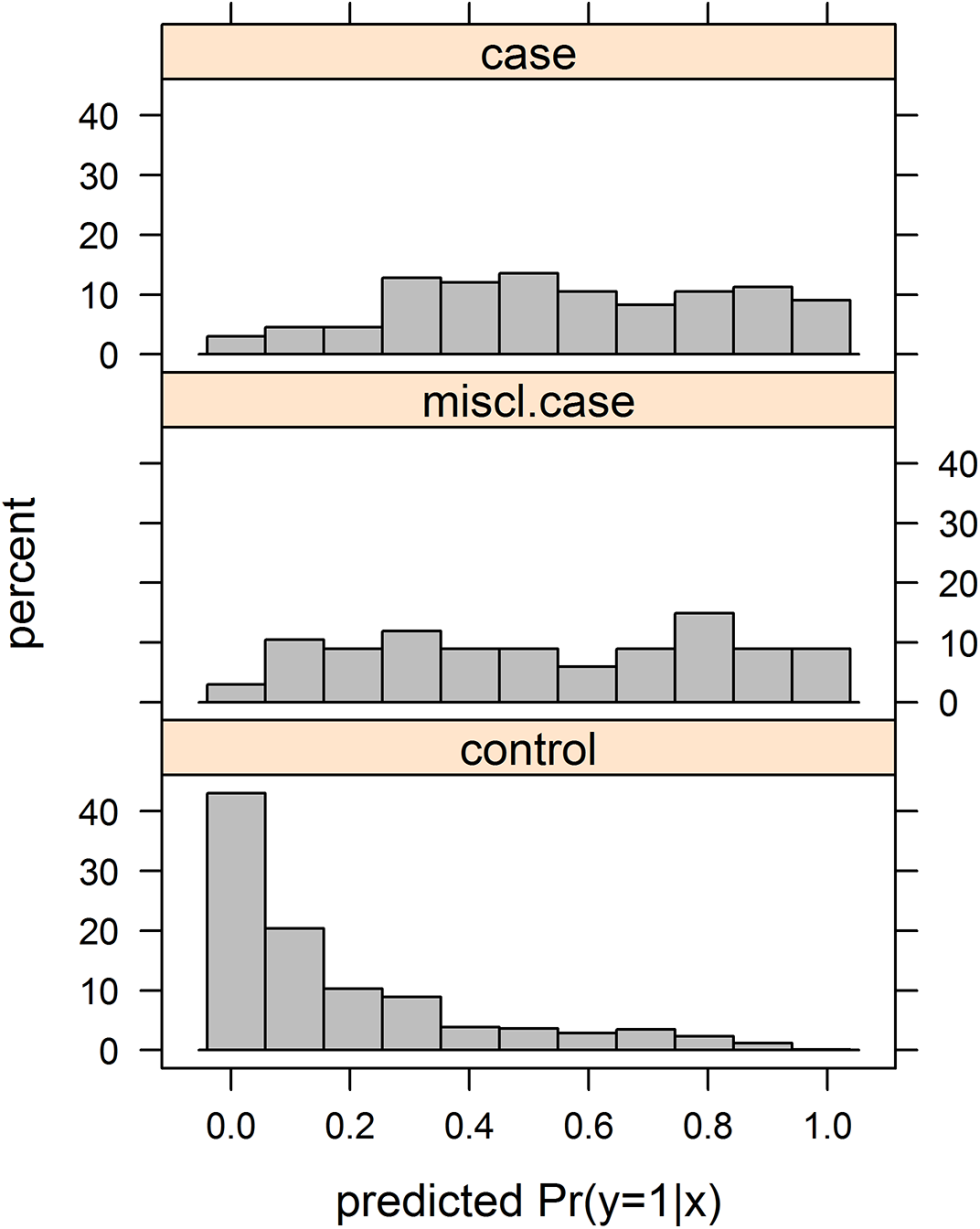
Frequency distribution of predicted probabilities of being a case in Example 2.

**Fig 3:**
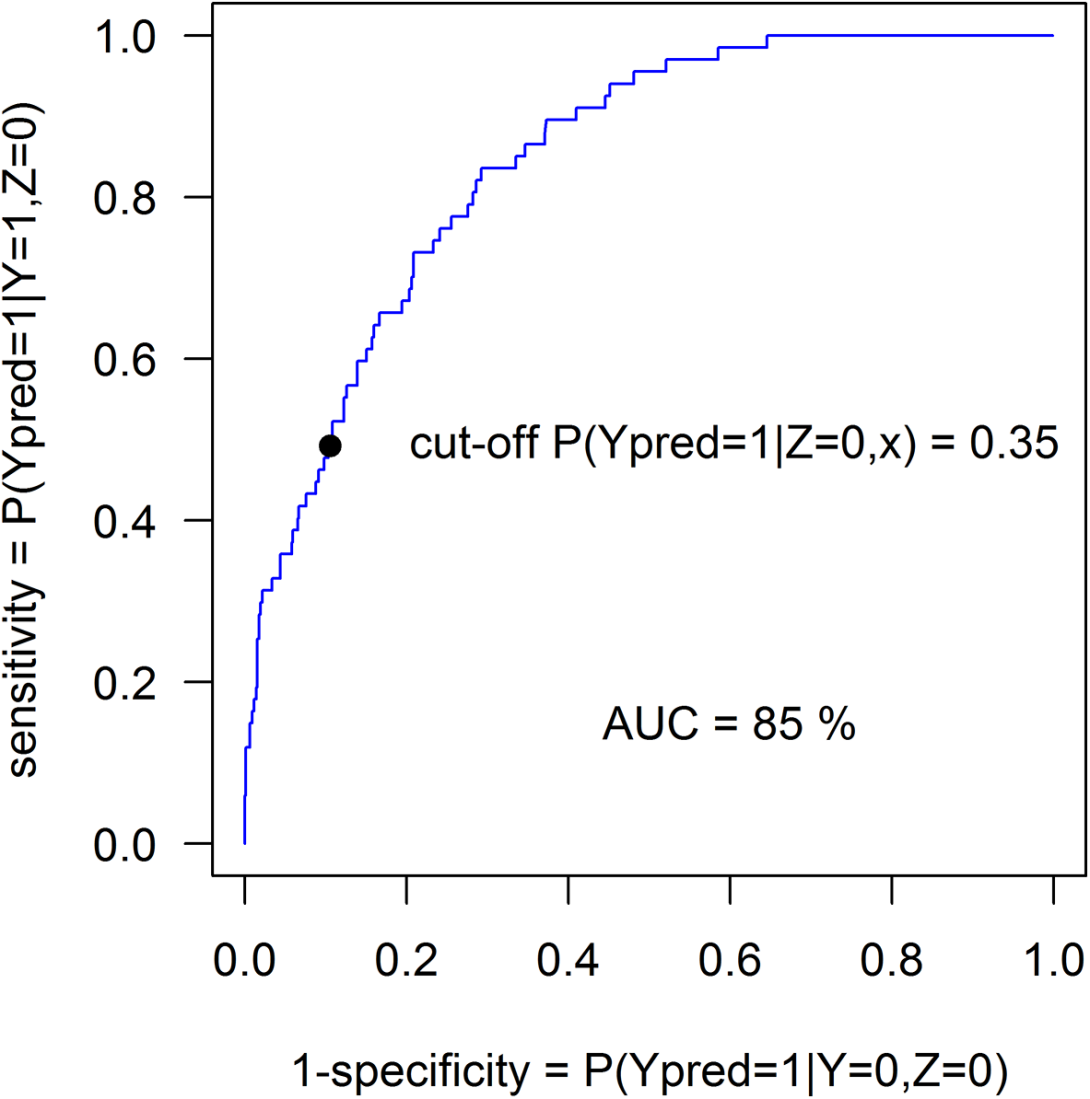
ROC curve for artificially mislabeled control group data of Example 2.

For our second example, to explore the DLR model in a situation in which the level of misclassification is (artificially) known, we used data from the Ille-et-Verlaine case-control study on esophageal cancer, with 200 cases and 778 controls (776 with complete data), by Tuyns *et al* [20] (data obtained from: http://faculty.washington.edu/norm/datasets.html, see S1 File). Four, highly significant (by standard logistic regression), covariables were of interest, age (rescaled), the square of age (age2), tobacco group (treated as a continuous variable) and daily alcohol consumption (in g/day, also rescaled). Applying DLR to these data correctly estimated λ = 0. We then intentionally randomly misclassified 67 cases as controls and used DLR to estimate the fraction misclassified. Table 3 and Table 4 summarize the data and the results of fitting the DLR and LR models. The ML estimate of the expected number of controls who are actually cases, n_1_ λ/(1-λ), is 116 (95% CI: 15–216). The real number 67 falls well within the CI. However, the assumed underlying logistic function may also not be entirely correct, and such violations of assumptions may bias estimates of λ/(1-λ). For example, it seems unlikely that the probability of esophageal cancer can really approximate 1, as all cases must have been non-cases prior to developing their disease, with the same covariable pattern (except perhaps for a slightly lower age). The P-value of likelihood ratio test comparing LR and DLR is 0.021, thus suggesting likely superiority of the DLR over the LR. Of course, as the hypothesis λ = 0 is on the boundary of the parameter space this P-value has to be taken with a grain of salt. Using the DLR estimates of β_0_ and β we calculated the predicted probabilities P(y = 1|**x**) of being a case. Histograms of estimated probabilities P(y = 1|x) are shown in Fig 2. As an example we chose a cut-off 0.35 for which 116 of 776 “z = 0” controls were classified as cases (116 is the estimate by the DLR model), that is the estimated probability P(y = 1|z = 0,x) exceeded the cut-off. Then 33 (49%) out of 67 misclassified cases and 694 (89%) out of 776 controls were classified correctly. The ROC curve is shown in Fig 3. To further explore the behavior of the proposed method we analyzed one hundred samples obtained by selecting randomly 67 misclassified cases. The median number of estimated misclassified controls was 103 (IQR: 69 to 142).

**Table 3:** Summary statistics of risk factors age, daily alcohol, and daily tobacco use, among esophagus cancer cases (n = 200) and controls (n = 776).

	Control	Case
Risk Factor	Mean (SD)	Mean (SD)
Age (years)	50.2 (14.3)	60.0 (9.2)
Alcohol (g/day)	44.3 (31.9)	85.1 (48.5)
Tobacco (g/day)	%	%
None	32.9	4.5
1–4	11.2	9.0
5–9	13.7	25.5
10–14	14.3	20.0
15–19	8.6	9.0
20–29	12.8	16.5
30–39	3.1	8.0
40–49	3.1	6.5
50-	0.4	1.0

**Table 4:** Parameter estimates and their standard errors of DLR and LR estimation

	DLR		LR	
variable	estimate	SE	estimate	SE
μ = λ/(1-λ)	0.869	0.385		
b_0_	-2.515	0.361	-2.953	0.248
b_(age-50)/10_	1.665	0.380	1.157	0.187
b^2^_(age-50)/10_	-0.526	0.153	-0.357	0.089
b_tobacco_	0.330	0.093	0.235	0.053
b_(alcohol-50)/10_	0.306	0.080	0.182	0.025
-2∙log(L)	589.23		594.54	

## Discussion

Misclassification is an important statistical problem that appears in many different contexts, including the estimation of discrimination and classification rules. Misclassification or mislabeling in training samples may seriously bias classifiers that are estimated from such samples. Ignoring it may lead to biased estimates of the β; generally biases will be towards the null.

We explored a special case in which misclassification occurs in one direction only in the context of logistic discrimination. It turns out that a simple modification of the standard logistic regression function, *viz*. a defective logistic regression function, can take such misclassification into account. Consistency, bias, and meaningfulness of estimators, however, depend of the (in)correctness of the underlying logistic discrimination function. Estimates of λ may also mostly depend on the fraction of observation with z = 0 but P(y = 1|x) close to 1, and small numbers of those cases might introduce bias. The effects of suspected model misspecification (*i*.*e*. of the logistic function) can be explored, on an *ad-hoc* basis, *e*.*g*. using computer simulations. Our method can easily be extended to other link functions. For example, in serological surveys to detect past exposure to an infectious agent, the use of a test (*e*.*g*. Elisa) with imperfect sensitivity will lead to zero-inflated complementary log-log link binomial regression.

Standard software should be extended with our proposed procedure.

## Supporting Information

S1 File Esophagus cancer case-control data. Both true case-control status y (1 = case, 0 = control) and intentionally misclassified (67 cases) status (z) are shown, in addition to the covariables age, tobacco use, and alcohol consumption.
(TXT)
